# Challenges and innovations in brain PET analysis of neurodegenerative disorders: a mini-review on partial volume effects, small brain region studies, and reference region selection

**DOI:** 10.3389/fnins.2023.1293847

**Published:** 2023-11-30

**Authors:** Prabesh Kanel, Giulia Carli, Robert Vangel, Stiven Roytman, Nicolaas I. Bohnen

**Affiliations:** ^1^Department of Radiology, University of Michigan, Ann Arbor, MI, United States; ^2^Morris K. Udall Center of Excellence for Parkinson’s Disease Research, University of Michigan, Ann Arbor, MI, United States; ^3^Parkinson’s Foundation Research Center of Excellence, University of Michigan, Ann Arbor, MI, United States; ^4^Department of Neurology, University of Michigan, Ann Arbor, MI, United States; ^5^Neurology Service and GRECC, Veterans Administration Ann Arbor Healthcare System, Ann Arbor, MI, United States

**Keywords:** reference region, partial volume effect correction, spatial resolution, small region, SWEDDs

## Abstract

Positron Emission Tomography (PET) brain imaging is increasingly utilized in clinical and research settings due to its unique ability to study biological processes and subtle changes in living subjects. However, PET imaging is not without its limitations. Currently, bias introduced by partial volume effect (PVE) and poor signal-to-noise ratios of some radiotracers can hamper accurate quantification. Technological advancements like ultra-high-resolution scanners and improvements in radiochemistry are on the horizon to address these challenges. This will enable the study of smaller brain regions and may require more sophisticated methods (e.g., data-driven approaches like unsupervised clustering) for reference region selection and to improve quantification accuracy. This review delves into some of these critical aspects of PET molecular imaging and offers suggested strategies for improvement. This will be illustrated by showing examples for dopaminergic and cholinergic nerve terminal ligands.

## 1 Introduction

Positron emission tomography (PET) is becoming more common in both research and clinical settings. PET images are particularly useful due to the use of radioactive tracers, called radioligands, for quantifying and visualizing *in vivo* biologic processes at the molecular level. In clinics, PET is used as an imaging modality to diagnose many diseases (Evangelista et al., 2021; Akamatsu et al., 2023). PET imaging can target specific molecules or processes using selected radiotracers, and with appropriate modeling, can provide various quantitative metrics to elucidate these human physiological and pathological processes (DeLorenzo et al., 2009; Miller-Thomas and Benzinger, 2017; Zhou et al., 2020; Kanel et al., 2022a,b; Kanel et al., 2023). The Distribution of Volume Ratio (DVR) is a widely used metric that compares the volume distribution of regions with receptors to those without receptors, commonly applied to molecular radiotracers targeting neurotransmission systems or abnormal proteins like tau and amyloid-β. DVR serves as a simplified reference tissue model and is applicable when there is a well-defined kinetic model for a specific radiotracer, assuming knowledge and application of equilibrium in the PET acquisition protocol. The arterial input model (multi-compartment modeling) is considered the gold standard for quantification of PET signals and should be applied for the initial stage of validation of radioligands when first brought to human use. However, because of the invasive nature of an arterial input line and possible side-effects, particularly in older, vulnerable populations, there is a need for more simplified, reference-based models (Turkheimer et al., 2012; Kanel et al., 2020; Nugent et al., 2020). There are a number of conditions that would qualify a reference region. One of the methods to identify qualified reference region is by using unsupervised cluster analysis. Another widely used metric, the standard uptake value ratio (SUVR), suffers in accuracy due to the variability of radiotracer kinetics and is limited by bias as much as, if not more than the DVR (Heeman et al., 2022). While PET imaging possesses notable strengths, such as its high sensitivity that enables the detection of minute amounts of a radiotracer, it also has a drawback: relatively low spatial resolution (>3 mm), especially when compared to Magnetic Resonance Imaging (MRI) (<1 mm). This spatial resolution is a function of PET instruments themselves (with some new model of ultra-high resolution (UHR) scanner available overcoming the problem) but also a function of signal-to-noise ratios of the radiotracers (Catana, 2019; Rausch et al., 2019; Van Sluis et al., 2019). This limited spatial resolution gives rise to the partial volume effect (PVE) (Bettinardi et al., 2014), a phenomenon that introduces quantification biases and can result in misleading interpretations.

This manuscript addresses the main challenges related to PET imaging, particularly focusing on the complexities linked with partial volume effect correction (PVC) methods and the definition of the reference region in highly selective radiotracers (^18^F-Fluoroetoxybenzovesamical (FEOBV) and PE2I). These challenges hold regardless of the quantification methods used (DVR/SUVR). We also explore potential solutions to these challenges, namely UHR techniques and data-driven methods to determine more suitable reference regions, and how these solutions can drive advancements in PET quantification, especially in the study of smaller brain regions. The manuscript concludes by showcasing practical examples from current clinical practice and research, demonstrating how more sophisticated analytical approaches to molecular PET imaging can be beneficial to solve current diagnostic challenges (Erlandsson et al., 2012).

## 2 Perspectives on partial volume correction methods: do we need them?

Plenty of PVC methods have been developed with the aim of reducing or controlling the PVE (Rousset et al., 2007; Bettinardi et al., 2014). PVC methods can be categorized as image-based or ROI-based, depending on whether they consider the entire image or only a portion of it. Image-based methods are more common, as they allow the creation of versatile PVC-corrected images (Bettinardi et al., 2014). For example, the well-known Müller-Gärtner (MG) method improves spatial resolution in brain PET images, especially in cortical regions, through MRI-based anatomical registration (Müller-Gärtner et al., 1992). While popular in neurodegenerative research, it does not address spill-in and spill-out effects in adjacent gray matter regions. Other image-based methods, like ‘iterative Yang’ (Erlandsson et al., 2012), overcome this limitation by producing voxel-based PVC images and effectively correcting PVE in gray matter regions. However, they estimate values directly from PET data, thus making them primarily suited for high-resolution tomography (Lu et al., 2021). Despite the availability of several approaches, it is important to note that findings on their effectiveness vary, and standardized guidelines for their usage are lacking.

Some studies demonstrate improved quantitative analyzes across various experimental designs (Brendel et al., 2015; Rullmann et al., 2016; Gonzalez-Escamilla et al., 2017; Yang et al., 2019; Lu et al., 2021; Schuster et al., 2022), while others suggest that PVC may reduce interpretability by introducing noise (Thomas et al., 2011; Högenauer et al., 2016; Schwarz et al., 2019). Contrary to this, alternative studies have demonstrated that applying PVC does not alter the outcomes in both cross-sectional (Okkels et al., 2023a) and longitudinal comparisons (Villemagne et al., 2011). Additionally, when different methods are applied to the same sample, the results exhibit significant heterogeneity (Greve et al., 2016; Shidahara et al., 2017), indicating a need for a more robust methodology. In certain situations, this lack of robustness may warrant forgoing PVC altogether to avoid introducing random noise and altering regional tracer uptake in an unpredictable way.

To better grasp the complexity surrounding whether PVC should be used, it is crucial to recognize that this decision may vary depending on the research question and experimental design. PVE becomes more pronounced when the size of the region of interest is small compared to the resolution of the PET camera (Hoffman et al., 1979). This issue is particularly evident in conditions like dementia, where extended atrophy further exacerbates PVE. Considering this, experimental designs that involve patients with significant atrophy (e.g., demented patients or those in advanced stages of neurodegenerative diseases) or imply potential changes in brain cortical thickness (longitudinal designs) could be ideal candidates to benefit from PVC approaches. The situation might change when dealing with cohorts of patients who do not exhibit severe atrophy (e.g., early-stage disease or healthy controls). Applying PVC might not be as beneficial in such cases and could introduce noise. This noise can result in increased variability in the data distribution, which cannot be solely attributed to the underlying condition being studied.

Another crucial factor in the decision whether to use PVC is the characteristics of PET radiotracers. Some radiotracers, like ^18^F-FEOBV PET (Okkels et al., 2023b), have high affinity and specificity for their target molecular mechanism. As a result, they tend to experience more controllable spill-over effects; signal spreading from a target region to surrounding areas and vice versa. However, certain radiotracers, like ^18^F-Flortaucipir and ^11^C-Pittsburgh Compound-B PET (unspecific white matter binding), suffer from high off-target binding (Matsubara et al., 2016; Gonzalez-Escamilla et al., 2017), and controlling spill-over effects becomes more challenging. Applying PVC approaches might be necessary in this scenario regardless of the experimental design or the patients involved.

Despite the ongoing lack of consensus on PVC approaches, it is evident that all methods carry the potential to introduce uncontrolled noise. The situation is particularly crucial in multicenter studies, where the utilization of various PET cameras with differing resolutions already poses a challenge to harmonization. These differences necessitate varied PVC approaches. As a result, applying PVC might compound disparities rather than alleviate them. Considering all the above, exploring alternative strategies to mitigate the PVE becomes essential. That said, considering the need for guidelines for PVC to improve data replicability and reliability, it becomes urgent to conduct methodological studies exploring different scenarios for various radiotracers. In the interim, researchers are encouraged to report results both with and without PVC, along with the details of the PVC method used and the rationale (Knudsen et al., 2020). By adopting these practices, we can enhance the evaluation of the PVC effect on results and pave the way for more reliable and interpretable PET quantification in neuroimaging studies.

## 3 Should we study small brain regions? Is it possible with new advancements in PET, MRI, and radiotracers?

While the study of system-level changes in neurodegenerative disorders holds immense value, the accurate quantification of specific volumes of interest remains a crucial aspect of research in this field. Precisely quantifying specific (small) brain regions can significantly contribute to understanding the brain areas involved in distinct neurodegenerative mechanisms. The field of neurology and radiology is about to experience a groundbreaking shift with the introduction of the new UHR brain PET scanner (Gaudin et al., 2018; Doyon et al., 2023). This advanced technology has the potential to revolutionize the way we study and diagnose neurological conditions such as neuro-oncology, epilepsy, dementia, cerebrovascular disease, and more. The UHR brain scanner boasts an unprecedented resolution that can accurately characterize brain regions that were previously indistinguishable without the use of MRI. With the use of pixelated detectors, the UHR brain scanner can reach a 1.25 mm isotropic spatial resolution, and reconstructed spatial resolutions ranging from 1.32 to 2.41 mm, and 3.96 to 7.22 mm for radial images at 1 cm, and 10 cm from the center of the system field of view (FOV) respectively, which is a significant improvement over the current state-of-the-art reference for brain PET imaging (Gaudin et al., 2018; Doyon et al., 2023). As a result, several brain regions, like thalamic subnuclei, inferior and superior colliculi, and red nuclei can now be identified in a PET visually in UHR images. While the UHR PET scanner, like any new high-end technology, will likely be costly and take some time to become widely available, its introduction presents a promising step forward toward further advancements in neuroimaging. Previously, visualization and parcellation of these small regions were possible only in MRI images. We generate these parcellations by combining high resolution MRI images with brain metabolism data produced using a radioligand with a high signal-to-noise ratio, like ^18^F-FEOBV, a vesamicol analog that selectively binds to vesicular acetylcholine transporter (VAChT) in the brain. With the complimentary information garnered from MRI and PET, along with newly developed anatomic parcellation atlases of subthalamic nuclei (Iglesias et al., 2018), the cerebellum (Diedrichsen et al., 2011), and brainstem (Iglesias et al., 2015), we can effectively study smaller regions (Bohnen et al., 2021; Kanel et al., 2022b; Okkels et al., 2023a). One such approach involves manipulating the volume of the target region, focusing solely on the core voxels within the specific area and excluding those at higher risk of being influenced by surrounding regions (Bohnen et al., 2021). This can be achieved through morphological erosion/thresholding techniques without altering the molecular image itself (Bohnen et al., 2021). Furthermore, whenever possible, working within the subject space can help preserve volume integrity and reduce the risk of PVE in small regions. One should be careful by studying only small regions with a sufficiently high number of surviving voxels above a set post-erosion number to ensure the robustness of the findings. Another possible solution to consider is using only those voxels that have binding within the high approximately 20% for that particular region for calculating DVR or SUVR. One of the most important benefits of studying small regions is the ability to detect early changes associated with many diseases. For example, one study capitalized on ^18^F FEOBV PET’s high signal-to-noise ratio to distinguish LBD from PD. Cholinergic terminal reductions were most severe in limbic regions in dementia patients compared with PD, and least severe in occipital regions (Okkels et al., 2023a). Findings like these demonstrate that by examining smaller, more specific regions, we can better differentiate between potentially ambiguous disease pathologies and arrive at diagnoses earlier and more accurately. This provides potential avenues for earlier diagnoses and a better understanding of disease progression, both of which can significantly impact research and clinical settings.

## 4 Why choosing appropriate reference region is so important: looking at perspectives on reference regions on dopamine transporter tracers

The emergence of highly specific dopamine transporter tracers has significantly enhanced our means of studying the organization of the presynaptic dopaminergic system. Earlier radioligands, such as β-CIT, had high affinity for dopamine transporter (27 ± 2 nM), but also high affinities for serotonin (3 ± 0.2 nM) and norepinephrine (80 ± 28 nM) transporters (Emond et al., 2008). Modification of the β-CIT molecule to make it more selective to dopamine transporter yielded PE2I, which has high affinity for dopamine transporter (17 ± 7 nM), and very low affinities for serotonin (500 ± 30 nM) and norepinephrine (>1,000 nM) transporters (Emond et al., 2008). While such high selectivity should allow the study of dopaminergic system changes in low-binding regions, much of the focus to date has been on high-binding regions such as the striatum, due to its importance in the clinical context of movement disorders. Unfortunately, this heuristic approach has led to the neglect of several low-binding regions where dopaminergic innervation might nevertheless contribute to functional organization. Indeed, evidence exists about dopaminergic innervation in all lobes of the cerebral cortex (De Keyser et al., 1989; Berger et al., 1991; Lewis et al., 2001; Nagano-Saito et al., 2017; van Kempen et al., 2022), the cerebellum (Barik and de Beaurepaire, 1996; Melchitzky and Lewis, 2000; Hurley et al., 2003; Locke et al., 2018; Qian et al., 2018; Flace et al., 2021; Cutando et al., 2022; Chen and Zhang, 2024), and limbic regions (Barili et al., 1998), which elevates the importance of re-evaluating quantification methodologies to advance our understanding of the whole-brain dopaminergic system.

In the domain of semi-quantitative analysis in dopaminergic presynaptic molecular imaging, the cerebellum is a frequently selected reference region due to its presumed minimal dopaminergic innervation. Among the earliest studies cited to justify the use of cerebellum as a reference region for imaging of PE2I derived radioligands include human post-mortem autoradiography work on ^125^I-PE2I (Hall et al., 1999) and PET imaging of ^11^C-PE2I in human and non-human primates (Halldin et al., 2003). Both studies failed to show appreciable signal within the cerebellum, and the latter also showed failure to displace ^11^C-PE2I binding via blocking using β-CIT and GBR-12909 competitive ligands within the cerebellum and cerebral cortex, but not within the striatum, leading the authors to conclude that PE2I binding in the cerebellum is predominantly non-specific. Later works on kinetic modeling of PE2I provided evidence to the contrary, with multiple studies showing that the cerebellum does not strictly meet the assumptions that underpin its use as a reference region (Jucaite et al., 2006; Hirvonen et al., 2008; Odano et al., 2012). Some studies used only the hemispheric cerebellum as a reference region, excluding the midline vermis due to the potential presence of dopaminergic innervation (Varrone et al., 2011). More recent post-mortem immunocytochemistry data in humans, however, suggest that the cerebellar hemispheres might also receive dopaminergic innervation (Flace et al., 2021), which calls into question the continued use of the cerebellum as reference region. Alternative low-binding reference regions, such as the occipital cortex, were considered for PE2I due to the low norepinephrine and serotonin transporter presence in those regions (Emond et al., 2008), but they suffer from the same flaws as the cerebellar reference regions, since the density of dopaminergic terminals in those regions cannot be quantified when they are used as a reference, and independent evidence suggests that dopaminergic innervation there might not be negligible (De Keyser et al., 1989; Berger et al., 1991; Lewis et al., 2001; Nagano-Saito et al., 2017; van Kempen et al., 2022).

Arterial sampling is considered the gold standard method for kinetic modeling for several radiotracers (Rissanen et al., 2015; Naganawa et al., 2020). Patients undergo serial blood sampling during the scan, then the obtained samples are analyzed to calculate the arterial input function (AIF) (Sari et al., 2018). Arterial sampling has been used for kinetic modeling in many studies involving several tracers, including PE2I (Jucaite et al., 2006), FEOBV (Petrou et al., 2014), DTBZ (Chan et al., 1999), and others. However, the use of arterial sampling to obtain an AIF is invasive, and can be particularly challenging for patients (Smart et al., 2023). Data driven approaches present a promising path forward for non-invasive quantification methods. While some approaches, like Basis pursuit (DeLorenzo et al., 2009) and supervised clustering (Jonasson et al., 2017), to determine better reference regions for PE2I have largely been unsuccessful, alternative approaches, such as the use of unsupervised clustering algorithms, have scarcely been attempted, and should be pursued more. Unsupervised clustering would classify voxels within a dynamic PET image based on similarity of their time activity curves, and these curve clusters can then be examined to identify better reference regions (Zbib et al., 2015). These methods could be used to identify regions within the cerebellar hemisphere, or even vermis that, anatomically, are regionally devoid of dopamine, and examine them for their utility as reference regions. Applications of unsupervised clustering have been used to examine differences in dopamine transporter binding in patients with parkinsonism (Suh et al., 2020), but its applications remain to be validated. When used prudently in conjunction with the high-selectivity of the PE2I radioligand, these data-driven methods might pave the way for better, less invasive, quantification of dopamine innervation in low-binding regions and would expand the range of questions we are able to ask about the dopaminergic system beyond the striatum.

## 5 Dogmas in clinical nuclear medicine using binary readings based on striatal-only binary readings: is a diagnosis of subject with evidence of motor parkinsonism but without evidence of dopaminergic degeneration (SWEDD) sufficient to exclude an α-syncucleinopathy etiology?

Nigrostriatal dopaminergic denervation is a major pathobiology in Parkinson disease (PD) (Obeso et al., 2017). Application of presynaptic nigrostriatal nerve terminal imaging in clinical trials has shown that up to 10% of persons clinically diagnosed as PD have a normal striatal uptake pattern and thus a SWEDD diagnosis (Erro et al., 2016). SWEDDs may have essential, dystonic, psychogenic tremor syndromes or other causes. Patients who meet criteria for the so-called body-first PD subtype may initially present with an abnormal cardiac MIBG scan and apparently normal neostriatal binding. These patients risk receiving a SWEDD diagnosis years prior to developing the asymmetric or caudate-to-putamen striatal denervation gradients that are hallmarks of PD (Durcan et al., 2023). Visually more subtle striatal dopamine binding changes in patients with so-called ‘ET-plus’ syndrome may also be at risk of a binary SWEDD misdiagnosis. About 20% of octogenarians have evidence of dementia with Lewy bodies (DLB). A subset of about 8–10% of pathology-confirmed patients may lack nigrostriatal denervation and in turn may be misdiagnosed as SWEDD. Furthermore, concomitant Alzheimer’s disease (AD) pathology, common in DLB (~50%), can contribute to SWEDD misdiagnoses. A recent study of ^123^I-FP-CIT SPECT imaging in patients with AD found reduced binding not in the neostriatum (putamen and caudate nuclei) but rather in areas targeted by the ventrotegmental-mesocorticolimbic pathways, namely the ventral striatum, including the nucleus accumbens, hippocampus, and cingulum, when compared to controls (Sala et al., 2021). These observations show that judging SWEDD status merely based on the neostriatum (putamen and caudate nuclei), could increase the risk of misdiagnosing patients with Lewy body parkinsonism. Furthermore, special attention should be paid to additional regions of interest such as the nucleus accumbens and the limbic dopaminergic projection areas innervated by the ventral tegmental area. Collectively, these findings suggest that the current common binary practice of interpreting neostriatal dopamine binding only as normal vs. abnormal may miss clinically relevant information in the assessment of not only PD, but also related Lewy body dementias.

## 6 Conclusion

In the last two decades, advanced MR imaging techniques, including perfusion-weighted imaging (PWI), functional magnetic resonance imaging (fMRI), magnetic resonance spectroscopy (MRS), and diffusion-weighted imaging (DWI), and several PET imaging agents that target numerous metabolic pathways in the brain have expanded the tools available for studying the nervous system in the normal state and in various diseased states. With these advancements, we must recognize that each tool comes with its own strengths and weaknesses, and that there is no “one-size-fits-all” approach to brain imaging. With the development of more selective tracers, like PE2I, it becomes necessary to consider using regions other than the cerebellum, or more selectively, specific regions within the cerebellum, based on the anatomical absence of dopamine nerve terminals, as reference, and use data-driven approaches like unsupervised clustering algorithms to identify more suitable regions. We must also exercise caution when using PVC. While effective in experiments involving patients with significant atrophy, PVC can introduce unnecessary noise in patients without atrophy and may not be necessary with images from radioligands with high level of affinity and specificity like ^18^F-FEOBV. With the arrival of UHR PET scanners, development of high level of affinity and specificity radioligands, and with techniques that increase resolution through combining MR and PET technologies, exploration of even smaller, more specific brain regions will become possible. Examination of these smaller regions could give way to faster, more accurate diagnoses, bolstering our understanding of disease progression and ensuring that clinically relevant information is not missed when assessing patients with neurodegenerative disorders. The field of neuroimaging is progressing rapidly, and with each advancement comes an opportunity to elevate our understanding of neurodegenerative diseases. We must exercise caution when using these tools, however, so that we maximize the accuracy and impact of our discoveries.

## Author contributions

PK: Writing – original draft, Writing – review & editing. GC: Writing – original draft, Writing – review & editing. RV: Writing – original draft, Writing – review & editing. SR: Writing – original draft, Writing – review & editing. NB: Writing – original draft, Writing – review & editing, Conceptualization, Funding acquisition.
